# Enhancing anti-neuroinflammation effect of X-ray-triggered RuFe-based metal-organic framework with dual enzyme-like activities

**DOI:** 10.3389/fbioe.2024.1269262

**Published:** 2024-04-19

**Authors:** Shurui Chen, Jinpeng Gao, Sen Lin, Haosen Zhao

**Affiliations:** ^1^ Clinical Research Center, Third Affiliated Hospital of Jinzhou Medical University, Jinzhou, China; ^2^ Department of Orthopedic, Third Affiliated Hospital of Jinzhou Medical University, Jinzhou, China

**Keywords:** nanoparticles, spinal cord injury, nerve regeneration, immunoengineering, neuroinflammation

## Abstract

Traumatic spinal cord injury (SCI), often resulting from external physical trauma, initiates a series of complex pathophysiological cascades, with severe cases leading to paralysis and presenting significant clinical challenges. Traditional diagnostic and therapeutic approaches, particularly X-ray imaging, are prevalent in clinical practice, yet the limited efficacy and notable side effects of pharmacological treatments at the injury site continue to pose substantial hurdles. Addressing these challenges, recent advancements have been made in the development of multifunctional nanotechnology and synergistic therapies, enhancing both the efficacy and safety of radiographic techniques. In this context, we have developed an innovative nerve regeneration and neuroprotection nanoplatform utilizing an X-ray-triggered, on-demand RuFe metal-organic framework (P-RuFe) for SCI recovery. This platform is designed to simulate the enzymatic activities of catalase and superoxide dismutase, effectively reducing the production of reactive oxygen species, and to remove free radicals and reactive nitrogen species, thereby protecting cells from oxidative stress-induced damage. *In vivo* studies have shown that the combination of P-RuFe and X-ray treatment significantly reduces mortality in SCI mouse models and promotes spinal cord repair by inhibiting glial cell proliferation and neuroinflammation. P-RuFe demonstrates excellent potential as a safe, effective scavenger of reactive oxygen and nitrogen species, offering good stability, biocompatibility, and high catalytic activity, and thus holds promise for the treatment of inflammation-related diseases.

## Highlights

• X-ray-triggered on-demand RuFe-based metal-organic framework (P-RuFe) are formed without an active pharmaceutical ingredient that can have offtarget effects in injured spinal cord.
• Following the combined strategy (P-RuFe and X-ray), immune cell infiltration is reduced, correlating with decreased tissue degeneration after SCI.
• The combined strategy developed into a permissive microenvironment characterized by proregenerative immune cell phenotypes, increased axons and myelination, and a substantially improved functional recovery.

## 1 Introduction

Spinal cord injury (SCI) is characterized as a debilitating condition resulting from spinal cord damage, profoundly impacting the physical, social, and vocational aspects of patient lives (Christopher et al., 2017a; Christopher et al., 2017b; Ilyas et al., 2021). Traumatic SCI, primarily caused by external physical forces such as vehicular accidents, falls, sports injuries, and assaults, is a significant etiological factor (Christopher et al., 2017a; Christopher et al., 2017b; Ilyas et al., 2021). This trauma typically results in the transection of long axons in spinal cord neurons, triggering a complex array of cellular and molecular events. These events encompass inflammation, neuronal damage, and cell death, often culminating in paralysis (John and Cristina, 2002). Effective therapeutic strategies for traumatic SCI hinge on two pivotal processes: the differentiation and activation of cells to foster neurite outgrowth for neuroregeneration, and the mitigation of inflammation for neuroprotection (Fan et al., 2018). However, current pharmacological interventions in clinical settings are limited, typically addressing either neuroprotection or neuroregeneration, but not both aspects of this multifaceted pathology. Furthermore, the presence of the blood-spinal cord barrier (BSCB) poses a significant challenge, impeding the efficient delivery of therapeutic agents to the spinal cord and thus diminishing their therapeutic potential (Christopher et al., 2017a; Christopher et al., 2017b; Ilyas et al., 2021).

Drugs with strong antioxidant effects and superior biocompatibility are required for effective treatment of SCI. Nanozymes possessing enzyme-mimetic activities have attracted substantial attention in the treatment of oxidative stress-related diseases, such as diabetic wounds, osteoarthritis, and SCI (Zheng et al., 2024). Advances in nanozyme development may introduce some methods for applying biomedicine to the treatment of intractable inflammatory diseases, including traumatic spinal cord injury (Jiang et al., 2023). Oxidative stress and inflammation are central pathophysiological processes in a traumatic spinal cord injury. Antioxidant therapies that reduce the reactive oxygen and nitrogen species (RONS) overgeneration and inflammation are proved promising for improving the outcomes (Wang Z. et al., 2022; Wu et al., 2023a; Chen et al., 2024). However, efficient and long-lasting antioxidant therapy to eliminate multiple RONS with effective neuroprotection remains challenging (Wu et al., 2023b; Wu et al., 2023c; Hou et al., 2024). Concurrently, X-ray-based radiographic imaging has established itself as a dependable modality for evaluating the severity of central nervous system disorders (Saito et al., 2011; Baaken et al., 2019). Recent advancements in multifunctional nanotechnology and synergistic therapeutic approaches have shown promise in augmenting both the efficacy and safety of X-ray applications in this context (Lin et al., 2016; Cheng et al., 2020; Liu et al., 2021). Consequently, there is an imperative demand for the development of an integrated, multifunctional drug delivery platform that addresses the complexities of SCI treatment.

Photobiomodulation therapy, an emerging treatment modality for various diseases, demonstrates efficacy in inflammation suppression through the application of high-energy ionizing beam radiation, including laser (Wickenheisser et al., 2019), X-ray (Wang Y. et al., 2022), and gamma-ray (γ-ray) (Kaliki and Shields, 2017). However, the absorption of high radiation doses by healthy tissues often results in inevitable damage to normal skin and organs, thereby constraining the advancement of this technique (Schaue and McBride, 2015; Allen et al., 2017). Over recent decades, significant efforts have been directed towards refining SCI photobiomodulation therapy for enhanced efficiency (Dompe et al., 2020; Ramezani et al., 2020). Despite these advancements, prolonged application of this strategy may still lead to damage in normal tissues. The incorporation of high atomic number (Z) elements in nanozyme-based strategies has garnered attention for their potential to augment radiation-induced therapeutic efficacy (Liu et al., 2018; Cao et al., 2020). High Z elements within nanozymes can act as radiosensitizers, leveraging their superior energy absorption capacity to enhance anti-inflammatory effects (Khurana et al., 2018; Hijmans et al., 2019). Nanoparticles containing high atomic number (high-Z) elements are recognized as superior agents for enhancing radiation sensitivity. Their effectiveness lies in their higher photoelectric absorption coefficient and the production of secondary electrons upon X-ray exposure, which significantly increases the radiation dose delivered to adjacent cells (Ebel et al., 2003). The Monte Carlo simulations have demonstrated that the heterogeneous distribution of high-Z nanoparticles, influenced by their sizes, can amplify secondary ionization throughout the interaction with X-rays. This amplification not only improves computed tomography (CT) imaging quality but also boosts the efficacy of radiation therapy (RT). Furthermore, the interaction of high-energy photons with high-Z metal nanoparticles generates Auger electrons (Zheng et al., 2021). This process enhance the activities of nanozymes.

**FIGURE 1:**
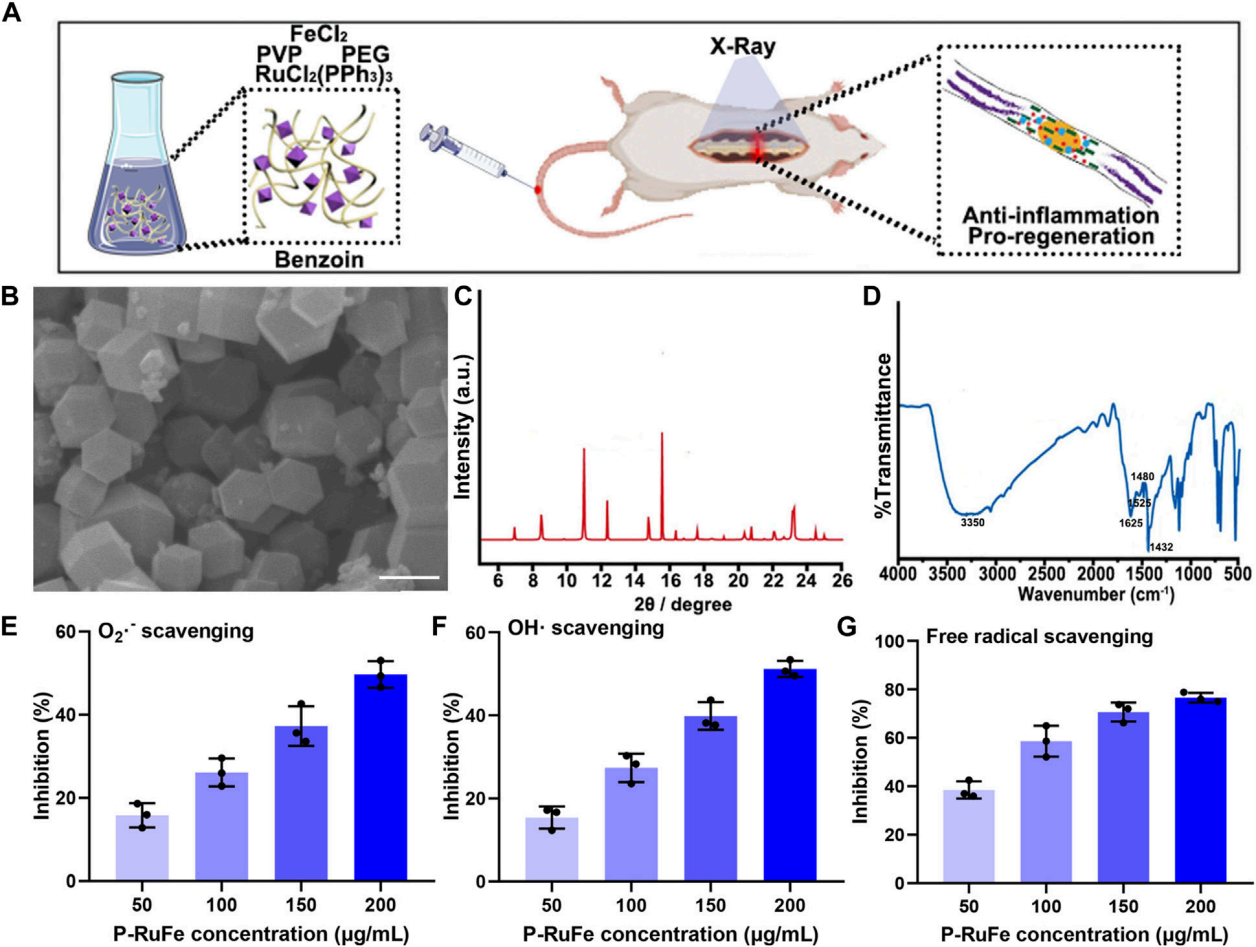
Characterization and antioxidant effect of P-RuFe. **(A)** Synthesis and mechanism of P-RuFe *in vitro* and *in vivo*. **(B)** SEM and size of P-RuFe. Scale bar is 200 nm. **(C)** PXRD of P-RuFe; **(D)** Infrared absorption spectroscopy comparison of P-RuFe. O_2_^−^ **(E)**,·OH **(F)** and free radical **(G)** scavenging ability of P-RuFe.

In this study, pegylated ruthenium-iron nanoparticles (P-RuFe) are introduced as a novel nano-sensitizer, aimed at improving the efficacy of photobiological regulation therapy for SCI. While numerous studies have focused on ameliorating the SCI microenvironment, particularly under hypoxic conditions closely linked to drug efficacy, a persistently high concentration of hydrogen peroxide (H_2_O_2_) remains a challenge. P-RuFe, exhibiting super-sensitive dual-enzyme-like properties, is capable of not only eliminating reactive oxygen species (ROS) such as H_2_O_2_ and superoxide (O_2_^−^), but also effectively scavenging reactive nitrogen species (RNS). Consequently, P-RuFe’s ability to protect cells from RNS-induced damage emerges as a potential therapeutic enhancement. Moreover, the high Z attribute of ruthenium (Ru) in P-RuFe facilitates active interaction with X-rays. By depositing higher energy, Ru maximizes the radiation dose at the lesion site, achieving a synergistically enhanced anti-inflammatory effect and significantly improving neuroprotection. P-RuFe has been observed to notably inhibit neuroinflammation and promote remyelination, increasing the sensitivity of photobiological regulation therapy. Additionally, this study demonstrates the feasibility of combining dual enzyme-like nanomaterials with radiosensitizer elements to alleviate SCI dysfunction and enhance anti-inflammatory responses, showing promise for the treatment of RNS-associated inflammation (Figure 1A).

## 2 Results and discussion

### 2.1 Synthesis and characterization of P-RuFe nanoparticles

The synthesis of RuFe nanoparticles, designated as P-RuFe, was conducted by reducing RuCl_2_(PPh_3_)_3_ and FeCl_2_ using ethylene glycol. Additives including benzoin and polyvinylpyrrolidone (PVP) were incorporated into the reaction mixture at 180°C. Following the reaction, the synthesized RuFe nanoparticles were isolated via centrifugation, with unreacted substrates subsequently removed through repeated washing and centrifugation. To enhance biocompatibility and water solubility, the nanoparticle surfaces were conjugated with methoxy polyethylene glycol amine (mPEG-NH_2_). The surface morphology of the mPEG-conjugated RuFe nanoparticles was characterized using scanning electron microscopy (SEM), as depicted in Figure 1B. The lateral dimensions of P-RuFe, approximately 200 nm, resulted in a distinct particle-like structure. Powder X-ray diffraction (PXRD) analysis of the nanoparticles showed a pattern consistent with the expected framework, indicating successful synthesis (Figure 1C). Infrared spectroscopy further confirmed the presence of P-RuFe, revealing the disassociation of organic molecules concurrent with framework formation (Figure 1D). The stretching vibration of RuFeMOF with peaks was 1,432, 1,480, 1,525, 1,615, and 3,350 cm^−1^, signify the primary structural constituents of the P-RuFe Nanoparticles we synthesized.

To evaluate the ROS scavenging capabilities of P-RuFe, three representative ROS were selected: O_2_^−^,·OH, and free radicals. Concentration-dependent ROS scavenging activity was observed, with 200 μg/mL of P-RuFe leading to approximately 50% decomposition of O_2_^−^ (Figure 1E). Similarly, at a concentration of 200 μg/mL, P-RuFe trapped over 50% of·OH (Figure 1F). The antioxidative properties of P-RuFe were further validated through a free radical scavenging assay using 2,2′-azino-bis(3-ethylbenzothiazoline-6-sulfonic acid) (ABTS). Consistent with previous findings, a low concentration of P-RuFe (200 μg/mL) effectively removed over 70% of free radicals (Figure 1G), demonstrating a dose-dependent radical scavenging efficacy.

### 2.2 RONS scavenging and cyto-protective effects of P-RuFe *in vitro*

**FIGURE 2:**
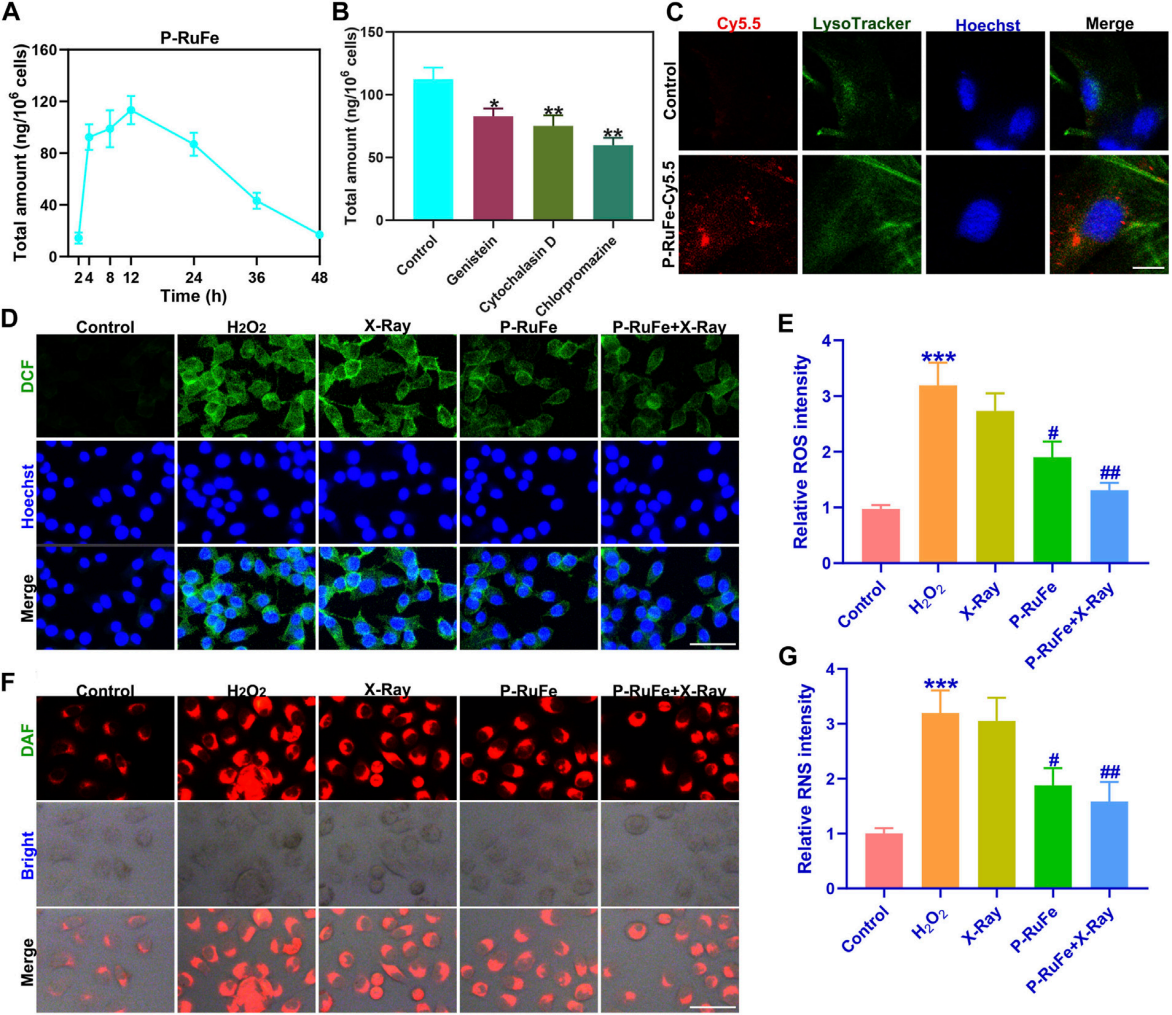
Cyto-protection of P-RuFe *in vitro*. **(A)** P-RuFe content in RAW264.7 cells following treatment with 100 μg/mL P-RuFe for the indicated periods of time. **(B)** To determine the accumulation of P-RuFe in RAW264.7 cells treated with 100 μg/mL of P-RuFe and various inhibitors of endocytosis for 12 h. Dosing: genistein, 50 μM; cytochalasin D, 10 μM; chlorpromazine, 20 μM. The content of P-RuFe was analyzed using ICP-MS. **(C)** Confocal images of RAW264.7 cells cells treated with 100 μg/mL P-RuFe-Cy5.5. **(D)** Representative ROS staining of RAW264.7 cells after different treatments over the course of 12 h. **(E)** Quantification of fluorescence of DCFH-DA probe (staining ROS) in RAW264.7 cells upon different treatments. **(F)** Representative RNS staining of RAW264.7 cells on different treatments over the course of 12 h. **(G)** Quantification of fluorescence of RNS in RAW264.7 cells upon different treatments. ***p < 0.001 compared with the Control group; #*p* < 0.05; ##*p* < 0.01 compared with the H_2_O_2_ group. Data are mean ± SD.

The critical importance of nanoparticle cellular internalization in achieving therapeutic outcomes is increasingly acknowledged. To study the cellular uptake of P-RuFe *in vitro*, an Agilent 7500 CE quadrupole ICP-MS (Agilent Technologies, Omaha, NE, United States) with a Micromist sprayer and a Scott Double Pass spray chamber was spent. P-RuFe treated after 2, 4, 8, 12, 24, 36, 48 h were tested in cells at a concentration of 200 μg/mL. This accumulation suggests effective internalization of P-RuFe within these cells (Figure 2A). To elucidate the potential mechanisms underlying P-RuFe cellular uptake, we investigated the effects of co-treatment with genistein, cytochalasin D, and chlorpromazine, known endocytosis inhibitors. Notably, both cytochalasin D and chlorpromazine markedly reduced P-RuFe nanoparticle uptake in RAW264.7 cells, as depicted in Figure 2B.

Further exploring cellular internalization, P-RuFe was labeled with Cy5.5, revealing its presence within endo/lysosomes post-treatment in RAW264.7 cells. This was evidenced by the co-localization of P-RuFe (red) and LysoTracker Green (green) stained endo/lysosomes, a phenomenon absent in untreated cells (Figure 2C). Given P-RuFe’s pronounced reactive oxygen and nitrogen species (RONS) scavenging capabilities, we next assessed its impact on cellular RONS clearance and cytoprotection by using the fluorescent probe 4,5-Diaminofluorescein diacetate (DAF-2 DA), which is specifically designed for the detection of nitric oxide (NO). A combination therapy involving P-RuFe and X-ray exposure led to the near-complete elimination of elevated ROS levels, returning the fluorescence intensity of H_2_O_2_-treated cells to levels comparable to untreated controls (Figures 2D, E). Similarly, this combined approach effectively scavenged intracellular H_2_O_2_-induced RNS (Figures 2F, G).

These findings suggest that the dual enzymatic activities of the P-RuFe and X-ray combination strategy can efficiently eradicate intracellular RONS, underscoring the potential of P-RuFe as an effective agent for intracellular RONS removal and cellular protection.

### 2.3 X-ray irradiation enhanced therapeutic efficacy of P-RuFe

**FIGURE 3:**
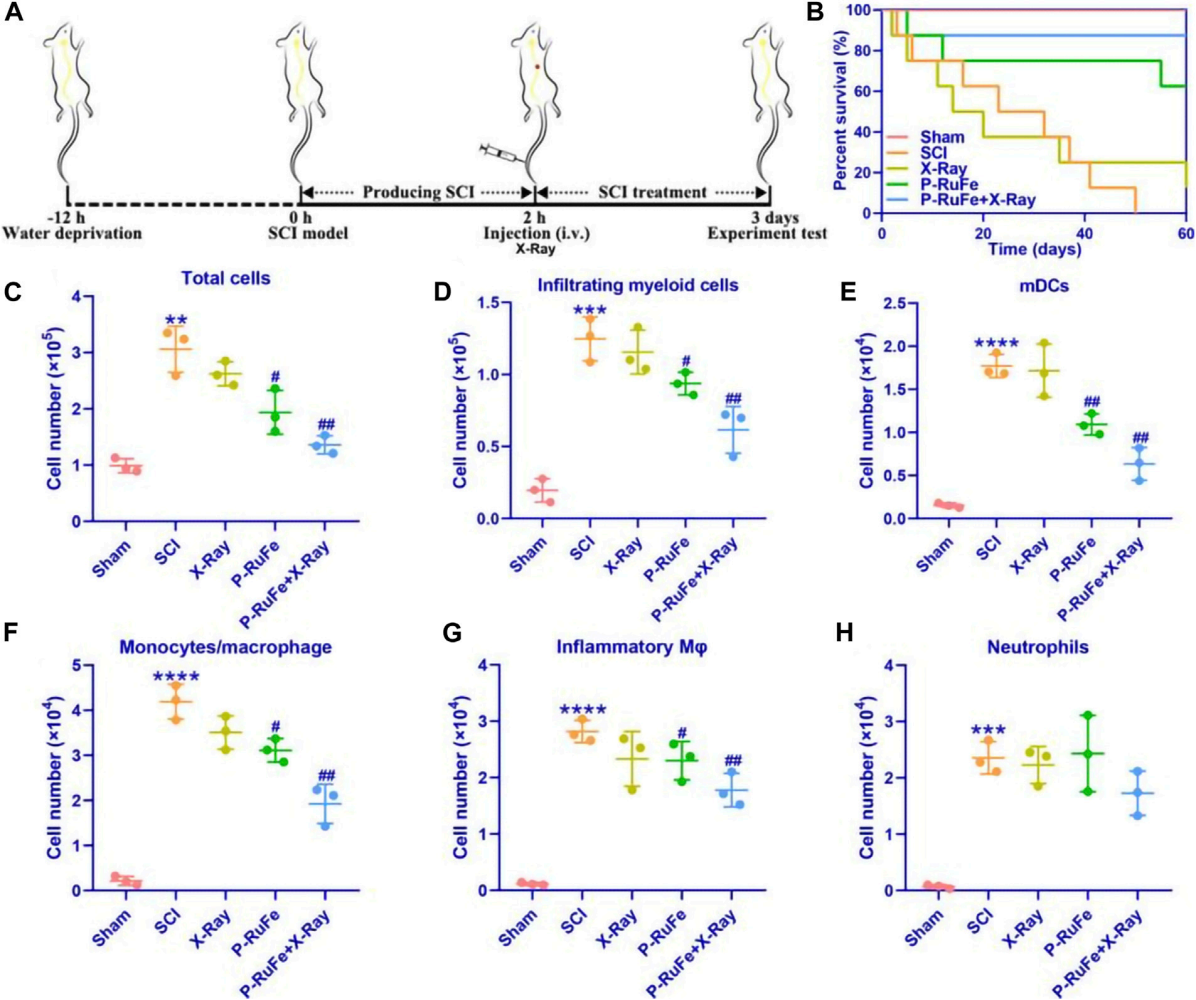
*In vivo* reduction of inflammatory cells infiltrating of combined strategy on Day 7 after SCI. **(A)** Schematic illustration of the establishment and treatment schedule of SCI mice. **(B)** Survival curves of SCI mice with different treatment. Representative quantification of total cells **(C)**, myeloid cells **(D)**, mDCs **(E)**, macrophages/monocytes **(F)**, inflammatory monocytes **(G)** and neutrophils **(H)**. **p < 0.01; ***p < 0.001; ****p < 0.0001 compared with the Sham group; #*p* < 0.05; ##*p* < 0.01 compared with the SCI group. Data are mean ± SD.

In a controlled *in vivo* study, adult mice subjected to a moderate contusion injury (200 kdyn) at the T9 vertebral level were used to establish a SCI model. Post-injury, the lesions were treated with P-RuFe nanoparticles and subjected to X-ray irradiation (Figure 3A). For the experimental setup, C57BL/6J mice were randomly assigned into five groups: (I) Sham surgery; (II) SCI; (III) SCI + X-ray irradiation (6 Gy); (IV) SCI + P-RuFe; and (V) SCI + P-RuFe and X-ray irradiation (6 Gy). Intravenous administration of saline (50 μL per mouse) or P-RuFe (5 mg/kg) was performed on days 0 and 3 post-injury, followed by X-ray treatment after 1 h where applicable.

As illustrated in Figure 3B, treatment with P-RuFe alone modestly improved the survival rate of SCI mice. However, the combined therapy of P-RuFe and X-ray irradiation significantly enhanced survival rates compared to other SCI-induced groups, indicating a substantial improvement in the therapeutic efficacy of P-RuFe. To elucidate the anatomical basis of this observed therapeutic effect, cells from the injury site were extracted 3 days post-SCI for flow cytometric analysis. A notable reduction in the total number of inflammatory cells was observed, particularly in the counts of CD45^hi^CD11b^+^Ly6G^−^CD11c^+^ myeloid dendritic cells (mDCs), CD45^hi^CD11b^+^Ly6G^−^CD11c^−^ macrophages/monocytes, and CD45^hi^CD11b^+^Ly6G^−^CD11c^−^Ly6C^hi^ inflammatory monocytes, in comparison to other groups (Figures 3C–G). However, no significant change was observed in the population of CD45^hi^CD11b^+^Ly6G^+^ neutrophils (Figure 3H). These findings collectively suggest that the combined treatment strategy effectively diminishes the infiltration of blood-derived inflammatory cells into the injured spinal cord during the acute phase of SCI.

Motivated by the pronounced anti-RONS effects of P-RuFe nanoparticles, and their role in enhancing photobioregulatory therapy, we explored the potential of P-RuFe as a radiation sensitizer in the treatment of SCI *in vivo*. Initially, CT imaging was employed to determine the severity of SCI in mice. Notably, P-RuFe treatment markedly enhanced the quality of CT images in SCI mice compared to those untreated, as shown in Supplementary Figure S1.

**FIGURE 4:**
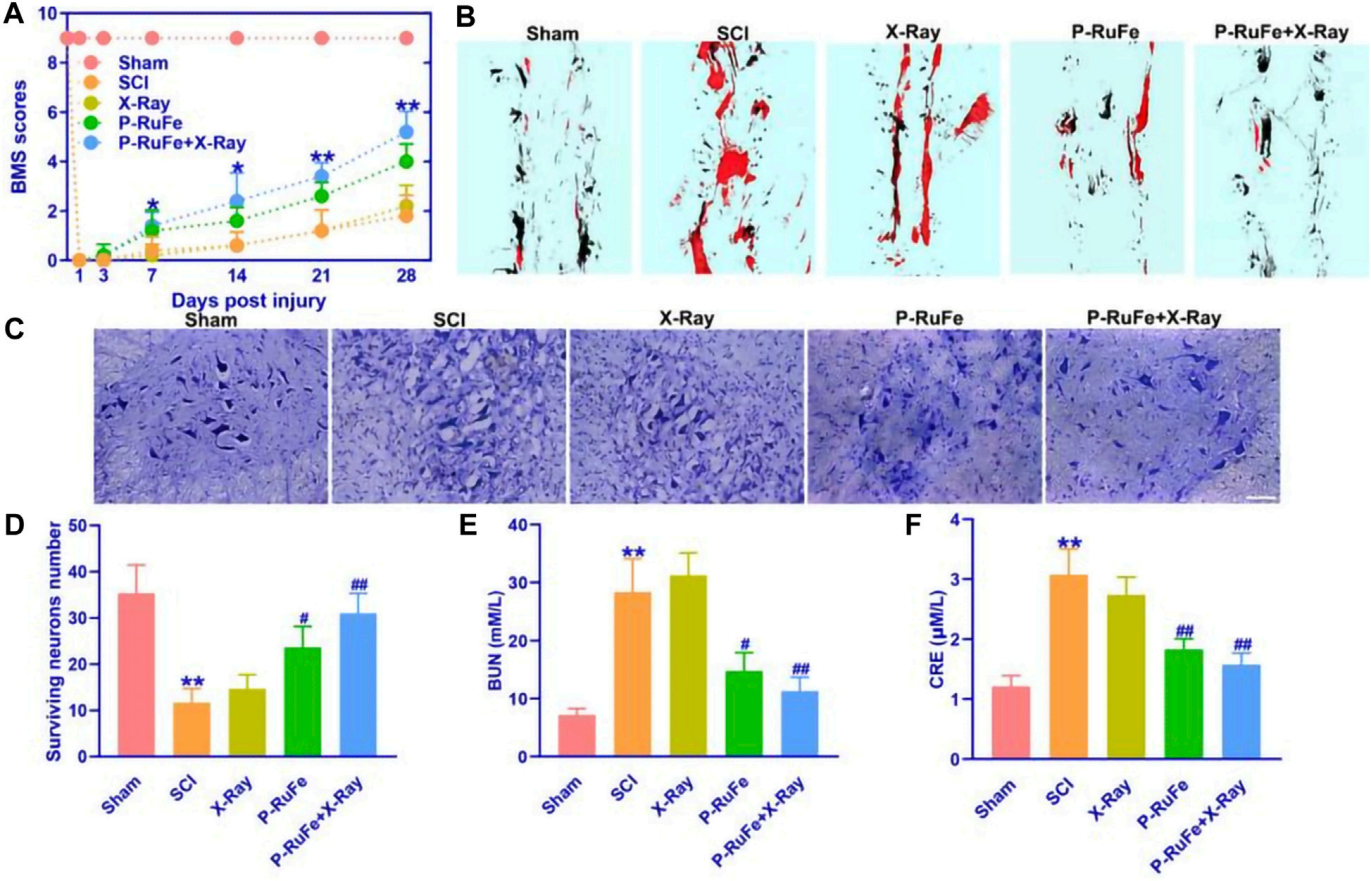
*In vivo* function recovery of combined strategy after SCI. **(A)** Representative quantification of the BMS scores of SCI mice subjected to different treatments. *p < 0.05; **p < 0.01 compared with the SCI group. **(B)** Representative quantification of gait experiment of SCI mice with different treatment. **(C)** Representative images of Nissl staining of SCI mice with different treatment. **(D)** Representative quantification of Nissl staining of SCI mice with different treatment. Representative quantification of BUN **(E)** and CRE **(F)**. **p < 0.01 compared with the Sham group; #*p* < 0.05; ##*p* < 0.01 compared with the SCI group. Data are mean ± SD.

To evaluate the therapeutic impact of P-RuFe *in vivo*, we conducted both the Basso Mouse Scale (BMS) for locomotion and gait analysis tests on SCI mice. In line with the anticipated therapeutic benefits, both BMS scores and hind leg gait functionality in SCI mice treated with P-RuFe + X-ray irradiation showed significant improvements (Figures 4A, B). Furthermore, to investigate the neuroprotective effect of P-RuFe, we analyzed the expression of neurons in the spinal anterior ventral horn (Figure 4C). Our findings revealed an increase in neuronal count in the P-RuFe-treated group compared to untreated SCI mice (Figure 4D). Interestingly, X-ray exposure did not significantly alter neuronal numbers in the P-RuFe group, affirming the neuroprotective efficacy of P-RuFe.

Additionally, to assess the biocompatibility of P-RuFe *in vivo*, we measured the levels of blood urea nitrogen (BUN) and creatinine (CRE) post-treatment (Figures 4E, F). Following the combination therapy of P-RuFe and X-ray irradiation, both BUN and CRE levels were observed to decrease, further substantiating the therapeutic effectiveness of P-RuFe. These results indicate that P-RuFe, with its notable biocompatibility and recovery functions, represents a promising approach for SCI treatment.

### 2.4 Macrophage polarization induced by P-RuFe plus X-ray at the injured lesion

The predominance of pro-inflammatory microglia/macrophages (M/Ms) within the SCI microenvironment impedes axonal regeneration and tissue repair. Consequently, modulating the activity of these pro-inflammatory M/Ms is crucial for therapeutic interventions. This study aimed to assess the impact of P-RuFe nanoparticles combined with X-ray irradiation on macrophage polarization, a key factor in neuroinflammation, within the SCI context.

**FIGURE 5:**
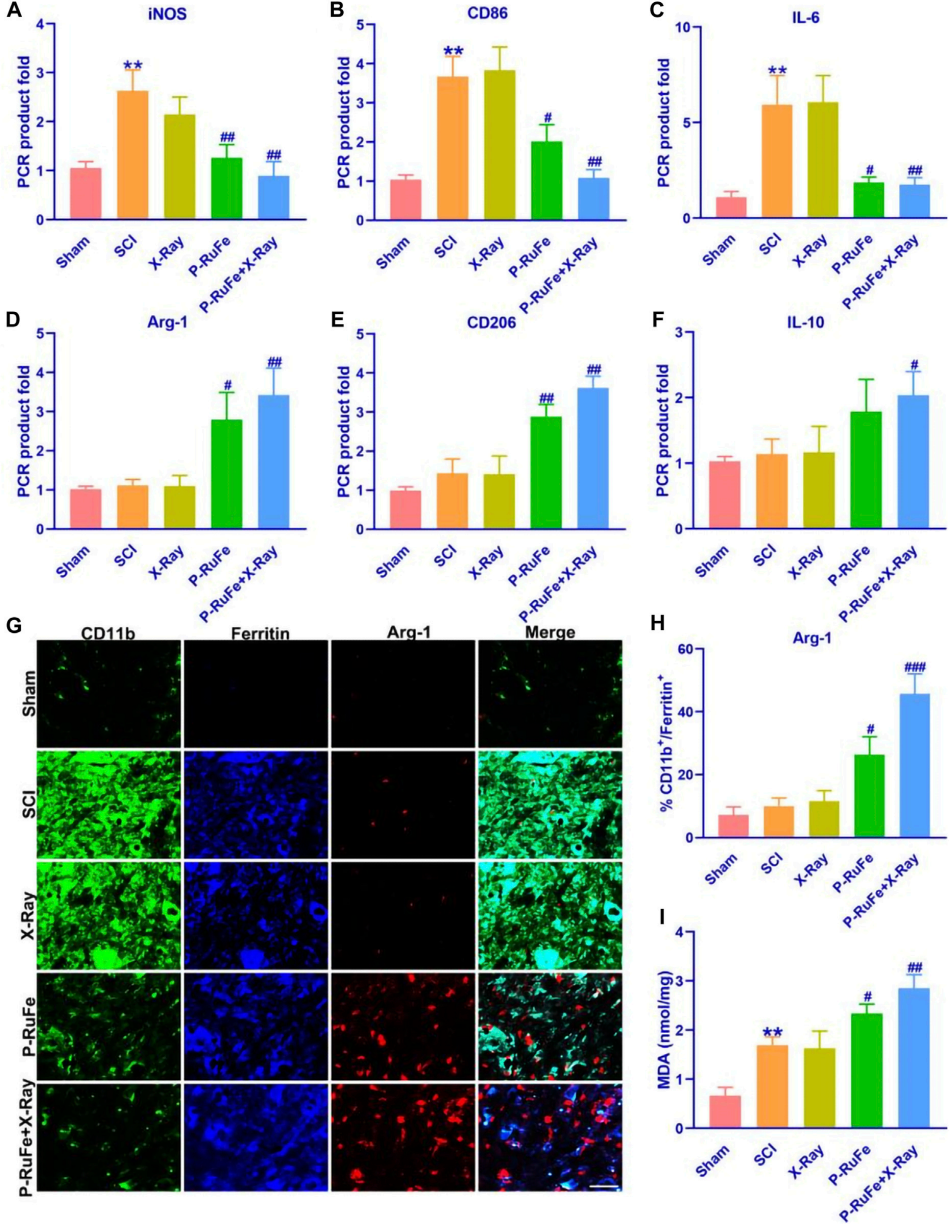
*In vivo* macrophages polarization of combined strategy. Representative quantification of mRNA levels of iNOS **(A)**, CD86 **(B)** and IL-6 **(C)**. Representative quantification of mRNA levels of Arg-1 **(D)**, CD206 **(E)** and IL-10 **(F)**. **(G)** Immunofluorescence images of Arg-1^+^, CD11b^+^,ferritin^+^ cells on 3rd day post-SCI. **(H)** Representative quantification of Arg-1^+^, CD11b^+^,ferritin^+^ cells on 3rd day post-SCI. **(I)** Representative quantification of MDA level on 3rd day post-SCI. **p < 0.01 compared with the Sham group; #*p* < 0.05; ##*p* < 0.01; ###*p* < 0.001 compared with the SCI group. Data are mean ± SD.

Our analysis focused on the expression of M1 and M2 macrophage markers in the injured spinal cord. We observed that the levels of inducible nitric oxide synthase (iNOS), CD86, and interleukin-6 (IL-6) mRNA—pivotal markers of the M1 phenotype linked to inflammation and pro-inflammatory cytokine release—were significantly reduced in the group treated with both P-RuFe and X-ray compared to other groups (Figures 5A–C). This reduction suggests an alleviation of the hyperinflammatory state at the lesion site.

Conversely, the expression of arginase-1 (Arg-1), CD206, and interleukin-10 (IL-10) mRNA, which are indicative of the M2 phenotype, was notably higher in the group receiving both P-RuFe and X-ray treatment compared to the groups treated with SCI and X-ray alone. However, no significant difference was observed between the group treated solely with P-RuFe and the one treated with both P-RuFe and X-ray (Figures 5D–F). These findings imply that P-RuFe can favorably modify the post-SCI microenvironment by inducing a shift in macrophage polarization at the injury site.

Spinal contusion injuries typically result in hemorrhage and the extravasation of red blood cells, which are swiftly phagocytosed by macrophages. This process may also lead to the release and subsequent macrophage uptake of iron from dying cells. Ferritin, a protein crucial for iron binding and storage, sees an upregulation in expression in response to increased intracellular iron levels, making it an effective marker for intracellular iron labeling.

Three days post-SCI, we detected the expression of tumor necrosis factor (TNF) in ferritin^+^ and CD11b^+^ microglia/macrophages (M/Ms). Notably, the expression of Arg-1 in iron-containing CD11b^+^ M/Ms was elevated by 42% and 25% in the P-RuFe + X-ray group and P-RuFe group, respectively (Figures 5G, H). These findings suggest a significant alteration in macrophage behavior and phenotype following treatment.

To further assess the oxidative stress response in macrophages post-treatment, we measured the levels of malondialdehyde (MDA), a marker of lipid peroxidation, using an enzyme-linked immunosorbent assay (ELISA) (Figure 5I). The MDA levels in groups treated with P-RuFe and X-ray were significantly higher than those in the SCI-only groups, indicating an enhanced oxidative stress response following these treatments.

### 2.5 Remyelination enhanced by P-RuFe plus X-ray in injured lesion

**FIGURE 6:**
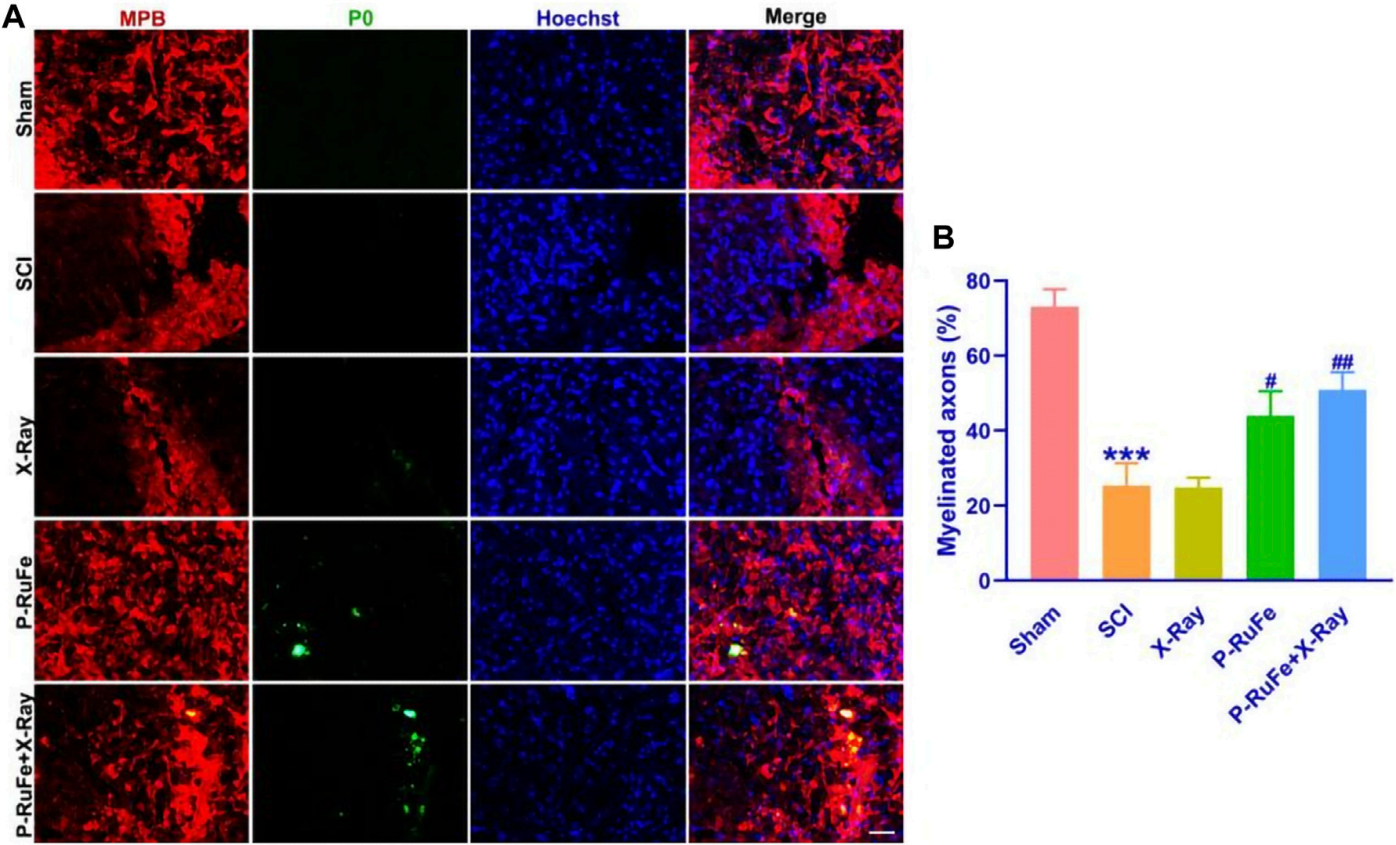
*In vivo* remyelination of combined strategy. Representative images **(A)** and quantification **(B)** of MBP^+^, P0^+^ cells on 28th day post-SCI. ***p < 0.001 compared with the Sham group; #*p* < 0.05; ##*p* < 0.01, compared with the SCI group. Data are mean ± SD.

In this study, we evaluated the regenerative potential of P-RuFe combined with X-ray irradiation during the early chronic phase of SCI, specifically 28 days post-injury (Figure 6). The assessment focused on the spinal cord, specifically labeling with myelin basic protein (MBP, red) and myelin protein zero (P0, green), key markers of myelin integrity and axonal health.

Our observations revealed that X-ray irradiation post-SCI facilitated gradual axonal regeneration. Notably, 28 days following treatment, the axonal ends in the P-RuFe-treated group were visibly regenerating. This regenerative effect was not evident in the group treated solely with X-ray (Figure 6A). Furthermore, the administration of P-RuFe in conjunction with X-ray significantly enhanced the number of myelinated axons compared to both the SCI-only and P-RuFe-only groups. The extent of remyelination in the combined treatment group was comparable to that observed in the undamaged contralateral side of the spinal cord (Figure 6B).

These findings indicate that P-RuFe, particularly when used in combination with X-ray irradiation, can effectively promote the repair of damaged neurons, offering a promising avenue for the *in vivo* treatment of spinal cord injuries.

## 3 Conclusion

In the current study, we have developed a novel metal-organic framework, P-RuFe, which is activated upon X-ray exposure. This framework exhibits dual functionality, encompassing both neurogenic and neuroprotective properties, thereby facilitating the repair of traumatic SCI through the amelioration of the post-injury microenvironment. Upon X-ray stimulation, P-RuFe demonstrates rapid intracellular uptake by lysosomes within macrophages, leading to a marked reduction in RONS levels.

Furthermore, *in vivo* experimentation revealed that a single X-ray irradiation in the presence of P-RuFe significantly enhanced the growth of motor neuron axons in mice models of traumatic SCI. This growth was accompanied by notable improvements in motor function rehabilitation. The underlying mechanism of P-RuFe’s efficacy appears to be its multifaceted ability to attenuate inflammatory responses and specifically target inflammatory macrophages.

Collectively, our findings not only underscore the potential of P-RuFe in nerve repair but also highlight its promising applications in the broader fields of neurological research and tissue engineering.

## Data Availability

The original contributions presented in the study are included in the article/Supplementary Material, further inquiries can be directed to the corresponding authors.
