# Supplementary figures and images for: Enhancing anti-neuroinflammation effect of X-ray-triggered RuFe-based metal-organic framework with dual enzyme-like activities
Source: Front Bioeng Biotechnol. 2024 Apr 19;12:1269262. doi: 10.3389/fbioe.2024.1269262 (PMC11066228; doi:10.3389/fbioe.2024.1269262)

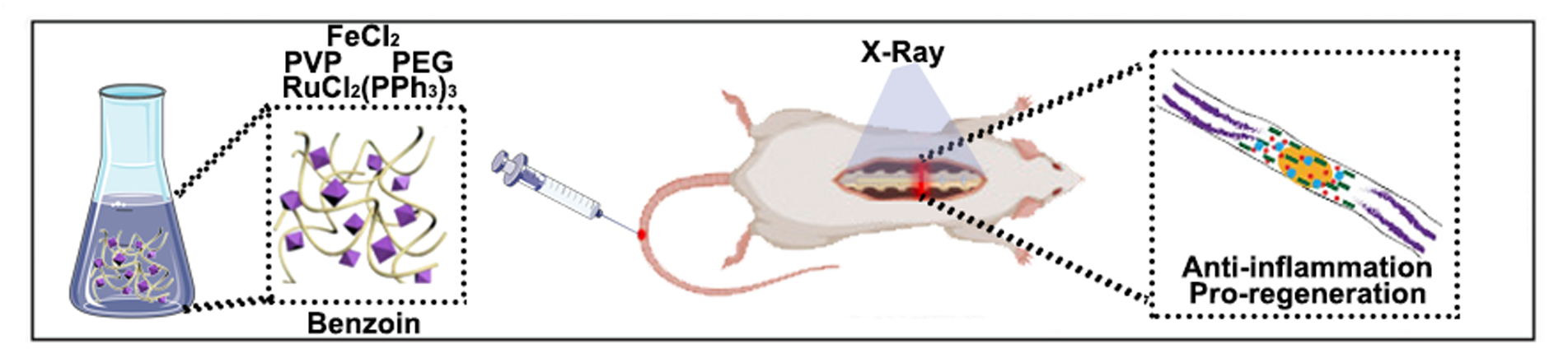

Supplement: Supplementary file 1 [file Image1.TIF]
