# Supplementary material for: Enhancing anti-neuroinflammation effect of X-ray-triggered RuFe-based metal-organic framework with dual enzyme-like activities
Source: Front Bioeng Biotechnol. 2024 Apr 19;12:1269262. doi: 10.3389/fbioe.2024.1269262 (PMC11066228; doi:10.3389/fbioe.2024.1269262)
Supplement: Supplementary file 2 [file DataSheet1.docx]

Supplementary material

**Enhancing anti-neuroinflammation effect of X-ray-triggered RuFe-based metal-organic framework with dual enzyme-like activities**

**This file includes:** Materials and methods, Supplementary Figure 1.

**Methods and materials**

**Synthesis of NPs**

Briefly, RuCl_2_(PPh_3_)_3_ (287 mg), FeCl_2_ (37 mg), benzoin (2.12 g) and PVP (10 g) were firstly dispersed in a mixture of 250 mL ethylene glycol. The mixture was stirred for 30 min and sonicated until the suspension became clear. Afterwards, the system was stirred at 180 ◦C for 5 h. After cooling to room temperature, the products were washed three times with an ethanol/acetone mixture and collected via centrifugation. Finally, the product was blow dried with nitrogen flow and stored in at 4 ◦C. To synthesize PEGylated Fe-modified Ru nanoparticles (P-RuFe), Fe-modified Ru nanoparticles (10 mg) as above were mixed with Milli-Q water (5 mL), then mPEG-NH_2_ (100 mg) was added into the solution, followed by sonicated for 30 min and stirred overnight. Subsequently, the solution was centrifugalized and washed three times with Milli-Q water to remove free mPEG-NH_2_. The final products were obtained by lyophilization and finally resuspended with Milli-Q water for further experiments.

**Animal treatments**

A total of 60 C57BL/6J mice (weight 18-24 g) were used. Mice were fed in a controlled place with standard rodents. Animals are kept at 22 ± 1°C, 12 hours light, 12 hours dark cycle. The study was permitted by the Jinzhou Medical University Review Board for the care of animals. A moderate contusion injury (200 kdyn) to T9 level of adult mice was made to generate the SCI in vivo model, P-RuFe and X-ray were applied to the lesion after SCI (Fig. 3a). C57BL/6J mice were divided randomly into five groups and the groups were as follows: (I) Sham group with saline; (II) SCI group with saline; (III) SCI group with X-ray (6 Gy); (IV) SCI group with P-RuFe; (V) SCI group with P–RuFe plus X-ray group (6 Gy). We carried out a 3-day treatment regimen with intravenous injection of either saline (50 μL/mouse), or P-RuFe (5 mg/kg) at day 0 and day 3 with/without X-ray treatments after 1 h.

**Behavioral Assessment**

The behavioral assessment was measured by behavioral analysis using the Basso Mouse Scale open-field locomotor test. A double-blind assessment was used at 0, 1, 3, 7, 14, 21, and 28 days post-injury. BMS scores range from 0 to 21 points. The 0 point reveals complete paralysis, and 9 points indicate normal function. The average scores were calculated by the grading standard in locomotion recovery after SCI. After surgery, the bladders were manually squeezed by applying pressure twice a day. We determine the relationship between pressure and urination ability, and grade bladder function from 0 to 3 (3 = dysfunction, high urination after the implementation of medium and high pressure; 2 = partial dysfunction, moderate urination after moderate pressure; 1 = mild dysfunction, squeeze out a small amount of urine after slight pressure; 0 = full function, no pressure required for urine discharge). This procedure continues until the bladder function of the mice recover.

**Quantitative real-time PCR (RT-qPCR)**

For quantitative real-time PCR (RT-qPCR) experiments, spinal cord tissue was obtained 7 days following injury. The total RNA was extracted using TRIzol reagent, and cDNA was synthesized from 5 μg total RNA. RT-qPCR was conducted using SYBR Green. Following are the conditions under which cDNA samples were amplified using a 7500 Rapid RT-PCR System (Applied Biosystems): 3 min at 95 °C followed by 40 cycles of 15 s at 95 °C and 45 s at 60 °C. In comparison of the target genes in experimental group, those in control group were applied with the method of (1+e) −ΔΔCT, and the corresponding expression levels of those target genes were normalized to ribosomal protein S18 (RPS18), the relative expression of the housekeeping gene. In order to normalize the level of mRNA in the samples, the amount of RPS18 in the samples was used (comparison of RNA levels with corresponding controls).

**Histological analysis**

The spinal cord tissues of mice were removed and fixed in 4% buffered formaldehyde for histological analysis. Afterwards, paraffin-embedded spinal cord sections were prepared, sectioned, DCF/ DHE stained, and ImageJ analyzed. We calculated results using the number of surviving neurons per 0.05mm^2^. For immunofluorescence staining, sections were blocked with 5% goat serum for 1 h at room temperature and then incubated with primary antibodies overnight at 4℃. The following day, Alexa Fluor-488 and Alexa Fluor-568 were used to stain the tissues for 2 hours at room temperature. Staining of the nuclei was performed using DAPI solution. 2mm range of images were obtained above and below the site of the spinal cord injury. Cell counts were performed using unbiased stereology. A positive cell rate per 0.05 mm^2^ was used to calculate the results. In order to calculate the absolute cell number counts and densities, the optical fractionator component of ImageJ2x software was utilized.

**Statistical Analysis**

Data are presented as mean ± SD and analyzed with SPSS 23.0. Student's t-test and one-way ANOVA determined data for two and more groups. Mann–Whitney U test was used for BMS analysis. The Wilcoxon rank-sum test was tested to compare data medians from quantitative PCR analysis and histogram analysis. P<0.05 was considered statistically significant.


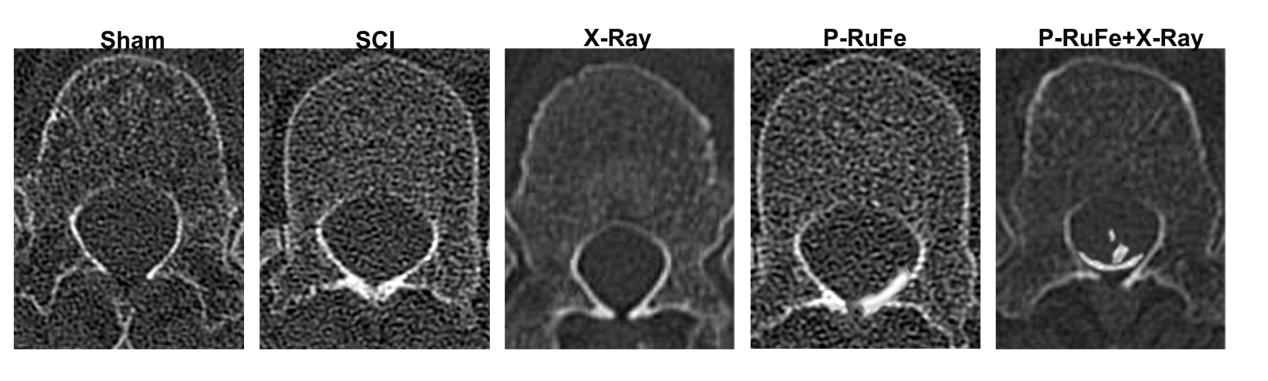


**Supplementary Figure 1.** Computed tomography images of injured spinal cord sections in vivo.
